# Efficacy of Combination Therapy with Oseltamivir Phosphate and Azithromycin for Influenza: A Multicenter, Open-Label, Randomized Study

**DOI:** 10.1371/journal.pone.0091293

**Published:** 2014-03-14

**Authors:** Hiroshi Kakeya, Masafumi Seki, Koichi Izumikawa, Kosuke Kosai, Yoshitomo Morinaga, Shintaro Kurihara, Shigeki Nakamura, Yoshifumi Imamura, Taiga Miyazaki, Misuzu Tsukamoto, Katsunori Yanagihara, Takayoshi Tashiro, Shigeru Kohno

**Affiliations:** 1 Department of Infection Control Science, Graduate School of Medicine, Osaka City University, Osaka, Japan; 2 Department of Molecular Microbiology and Immunology, Nagasaki University Graduate School of Biomedical Sciences, Nagasaki, Japan; 3 Divison of Infection and Control and Prevention, Osaka University Hospital, Osaka, Japan; 4 Nagasaki University Infection Control and Education Center, Nagasaki University Hospital, Nagasaki, Japan; 5 Department of Laboratory Medicine, Nagasaki University Hospital, Nagasaki, Japan; 6 Department of Health Sciences, Nagasaki University Graduate School of Biomedical Sciences, Nagasaki, Japan; University of Ottawa, Canada

## Abstract

**Background:**

Macrolides have antibiotic and immunomodulatory activities, which may have a favorable effect on the clinical outcome of patients with infections, including influenza. This study aimed to evaluate the effects of combination therapy with an anti-influenza agent, oseltamivir, and a single-dose formulation of azithromycin (AZM), which has been used for influenza-related secondary pneumonia, on influenza patients. The primary endpoint was a change in the expression levels of inflammatory cytokines. Secondary endpoints were the time required for resolution of influenza-related symptoms, incidence of complications, and adverse reactions.

**Methods:**

Patients with seasonal influenza were enrolled in this multicenter, open-label, randomized study. Patients were stratified according to the presence of a high risk factor and were randomized to receive combination therapy with oseltamivir plus an extended-release formulation of AZM (combo-group) or oseltamivir monotherapy (mono-group).

**Results:**

We enrolled 107 patients and randomized them into the mono-group (56 patients) or the combo-group (51 patients). All patients were diagnosed with influenza A infection, and none of the patients had comorbid pneumonia. Statistically significant differences were not observed in the expression levels of inflammatory cytokines and chemokines between the 2 groups. The maximum temperature in the combo-group was lower than that in the mono-group on day 3 through day 5 (p = 0.048), particularly on day 4 (p = 0.037).

**Conclusion:**

To our knowledge, this is the first prospective, randomized, clinical trial of oseltamivir and AZM combination therapy for influenza. Although the difference in inflammatory cytokine expression level was not statistically significant, combination therapy showed an early resolution of some symptoms.

**Name of registry:**

University hospital Medical Information Network (UMIN).

**Trial Registration no:**

UMIN000005371

## Introduction

Influenza virus infection is a major respiratory infectious disease that generally induces bronchitis, and occasionally leads to fatal pneumonia in the elderly when bacterial infections are involved [1]. Comorbid or secondary bacterial pneumonia is a severe complication related to the influenza virus infection, which suggests the importance of the latter infection in the morbidity and mortality in elderly patients with this disease [1], [2]. High mobility group B1 (HMGB1), a known proinflammatory cytokine and cytotoxic factor, is suggested to be involved in the development of influenza-related pneumonia [3]. In addition, increase in the levels of proinflammatory cytokines and monokines, including interleukin 1 (IL-1), IL-6, and IL-8, have been observed in the sera of patients and in the lungs of mice infected with the influenza virus [4]. These factors are suggested to be associated with the pathogenesis and severity of influenza virus infection [5].

Azithromycin (AZM), a 15-membered ring macrolide, is an azalide and is structurally related to the macrolide family of antibiotics. It binds to the 50S ribosomal subunit of susceptible organisms, thereby interfering with protein synthesis. AZM is approved worldwide as a broad-spectrum antibiotic for the treatment of a variety of community-acquired infections. A recently developed novel microsphere formulation of AZM (Zithromax SR 2 g) enables oral administration of high doses of AZM as a part of a single-dose regimen while maintaining tolerability.

Macrolides, including AZM and clarithromycin (CAM), a 14-membered ring macrolide, exert immunomodulatory effects on the host and antibacterial effects against the targeted microorganisms [6].

Viasus et al. reported that immunomodulatory therapies using corticosteroids and macrolides did not prevent the development of severe disease in patients with pandemic influenza A (H1N1) 2009 infection complicated by pneumonia [7]. Similarly, macrolide-based treatment has not been associated with improved survival in critically ill H1N1 patients with primary pneumonia in an intensive care unit (ICU) setting [8].

However, for patients with mild influenza, the duration of cough in patients without cough at the onset of pyrexia is significantly shorter with combined therapy with CAM and oseltamivir (Tamiflu) than that with oseltamivir monotherapy [9]. In addition, Kido et al. reported that while administration of CAM to influenza A virus (IAV)-infected mice decreases the production of tumor necrosis factor alpha (TNF-α) and increases the production of IL-12 in the blood, which results in the alleviation of flu symptoms [10], oral treatment with oseltamivir attenuates the induction of respiratory anti-IAV-specific secretory immunoglobulin A (S-IgA) immune responses [11]. Furthermore, a recent study showed that oral CAM increases the nasopharyngeal mucosal immune responses in IAV-infected children, while oseltamivir suppresses the production of mucosal anti-IAV S-IgA [12].

AZM may thus modulate airway inflammation induced by influenza virus infection. Basic studies have shown that AZM is effective against secondary bacterial pneumonia after influenza virus infection because of its inhibitory effect on the expression of various cytokines and its antibacterial activity [13].

In this study, we evaluated the efficacy of combination therapy with an anti-influenza agent, oseltamivir, and a single administration of an extended-release formulation of AZM and compared it with the efficacy of oseltamivir monotherapy in patients with influenza.

## Methods

The protocol for this trial and supporting CONSORT checklist are available as supporting information (see Checklist S1 and Protocol S1).

### Participants

We enrolled patients with influenza from the Nagasaki University Hospital and 13 of its affiliated hospitals and clinics. Patients aged 20 years and older with influenza A or B virus infection diagnosed by a positive rapid antigen test (RAT) for influenza were considered for enrollment. Patients had to have signs or symptoms of a seasonal flu or influenza A (H1N1) pdm 2009 virus infection with an axillary temperature ≥38.0°C and at least 2 of the following signs or symptoms at a moderate-to-severe degree: headache, muscle or joint pain, fever or chills, fatigue, cough, sore throat, and nasal stuffiness caused by influenza.

In addition, patients had to have accepted the treatment within 48 h from the onset of influenza symptoms, which were defined as follows: initial temperature elevation ≥1°C from the patient's normal body temperature or experience of at least 1 symptom included in the Influenza Symptom Severity scale (ISS) [14].

Patients with a history of hypersensitivity to AZM or oseltamivir and patients with bacterial infections were excluded. At screening, a complete history was recorded from all patients, including notes on flu vaccination, physical examination, chest radiographs, and blood chemistry. Assessment of clinical symptoms of influenza, including vital signs (body temperature, blood pressure, and pulse rate), was performed at baseline (day 0) and on days 2 and 5. Blood samples were collected on days 0, 2, and 5 for measurement of the levels of inflammatory cytokines and chemokines, HMGB1, and procalcitonin (PCT). Patients recorded their own influenza symptoms, maximal temperature, and activities of daily living using a 7-symptom ISS and a visual analogue scale (Influenza Impact Well-Being Score [IIWS]) ranging from 0 to 10.

### Study design

This prospective, randomized, open-label, controlled, multicenter study was performed between December 2010 and March 2011.

### Ethics

The trial was conducted in accordance with the Declaration of Helsinki and in compliance with the ethical guidelines for clinical studies issued by the Health, Labour and Welfare Ministry. Written informed consent was obtained from all patients before enrollment. The protocol, amendments, and informed consent documentation were approved by the institutional review board and/or independent ethics committee at each facility.

The project approval date for each Research Ethics Board is listed in brackets: Nagasaki University (October 13, 2010), The Japanese Red Cross Nagasaki Genbaku Isahaya Hospital (December 24, 2010), The Japanese Red Cross Nagasaki Genbaku Hospital (October 25, 2010), Hokusho Central Hospital (November 16, 2010), Sasebo General Hospital (January 17, 2011), NHO Ureshino Medical Center (November 22, 2010), Koseikai Hospital (November 22, 2010), Isahaya Health Insurance General Hospital (Not approved until the end of this study), Nagasaki Municipal Hospital (November 4, 2010), Onitsuka Naika Clinic (October 13, 2010), Hayashida Naika Clinic (March 12, 2011), Tomonaga Naika Clinic (October 13, 2010), Irihune Clinic (October 13, 2010), Kawamura Clinic (October 13, 2010).

The trial was first approved in October, 2010 by the ethics committee of Nagasaki University (accession number, 100100130), but was only finally approved by the other branch hospitals in March, 2011. Additionally, the trial was registered in the University Hospital Medical Information Network (UMIN) Center system. The UMIN accession number is UMIN000005371. The trial began in December, 2010.

### Study intervention and randomization

Patient enrollment was performed using a central registration system through a computer-generated random listing of the two treatment allocations. A minimization method [15] was used to randomize patients in a 1∶1 ratio to receive oral oseltamivir 75 mg alone (mono-group) every 12 h or oral oseltamivir 75 mg every 12 h in combination with an extended-release formulation of single-dose oral AZM 2,000 mg (combo-group). For randomization, patients were stratified according to the presence of high risk factors such as age (≥65 years), underlying respiratory diseases (e.g., chronic obstructive pulmonary disease, bronchial asthma), use of steroids (equivalent to prednisolone >10 mg/day), and uncontrolled diabetes mellitus (hemoglobin A1c [HbA1c] level >7.4; national glycohemoglobin standardization program [NGSP]). Oral oseltamivir was to be administered for 5 days in both groups.

### Outcome measures

The purpose of this study was to evaluate the efficacy and safety of combination therapy with an anti-influenza agent, oseltamivir, and AZM in patients with influenza.

The intent-to-treat (ITT) population was used prospectively for analysis. The ITT population included all patients who received 1 or more doses of the study medication. The primary endpoint was defined as variations in the levels of inflammatory markers (i.e., inflammatory cytokines and chemokines, HMGB1, PCT). Secondary endpoints were defined as follows: (1) the duration of influenza; (2) the incidence of influenza-related complications (sinusitis, otitis media, bronchitis, and pneumonia); (3) the time to alleviation of influenza symptoms; and (4) adverse events and adverse drug reactions.

### Inflammatory marker assays

The levels of the cytokines IL-1β, IL-2, IL-4, IL-6, IL-8, IL-10, IL-12, TGF-β, interferon γ (IFN-γ), and TNF-α were measured using the cytokine bead array.

### Clinical laboratory tests

We performed hematological (measurements of red blood cell [RBC] count, Hb level, hematocrit [Ht] level, platelet count, white blood cell [WBC] count, and WBC fraction); biochemical (measurement of the levels of aspartate aminotransferase [AST], alanine aminotransferase [ALT], total bilirubin [T-Bil], blood urea nitrogen [BUN], creatinine [Cre], total protein [T-P], albumin [Alb], sodium [Na], chloride [Cl], and potassium [K]); and immunological (measurement of C-reactive protein [CRP] level) tests on days 1, 2, and 5.

The differences in values on day 2 (ΔDay2) and day 5 (ΔDay5) from those observed on day 0 were evaluated.

### Statistical methods

The statistical analyses were performed by an expert biostatistician experienced in the subject studied. The mono-group included 56 patients, while the combo-group included 51 patients, which was the number estimated as the appropriate sample size (see the protocol).

This was an exploratory trial to assess the efficacy of oseltamivir plus AZM in the treatment of patients with influenza. Statistical analyses were performed using PASW Statistics 18 (SPSS Inc., Chicago, IL, USA). All tests were two-tailed, and a p value <0.05 was considered statistically significant. The significance of differences in the expression levels of cytokines and chemokines and in influenza-related symptoms between the mono-group and combo-group were examined using the Mann–Whitney *U* test. In addition, significance of differences in the maximum temperature between the mono- and combo-groups on days 3 through 5 were using a mixed-design analysis of variance (mixed-design ANOVA).

## Results

### Study population

A total of 107 patients were enrolled in the study between December 2010 and March 2011.

The number of patients enrolled at each hospital was as follows: 4, Nagasaki University; 12, The Japanese Red Cross Nagasaki Genbaku Isahaya Hospital; 8, The Japanese Red Cross Nagasaki Genbaku Hospital; 2, Hokusho Central Hospital; 6, Sasebo General Hospital; 6, NHO Ureshino Medical Center; 5, Koseikai Hospital; 0, Isahaya Health Insurance General Hospital (Not approved until the end of the study); 6, Nagasaki Municipal Hospital; 20, Onitsuka Naika Clinic; 0, Hayashida Naika Clinic; 20, Tomonaga Naika Clinic; 13, Irihune Clinic; and 5, Kawamura Clinic. All patients were recruited after the relevant project approval date.

**Figure 1:**
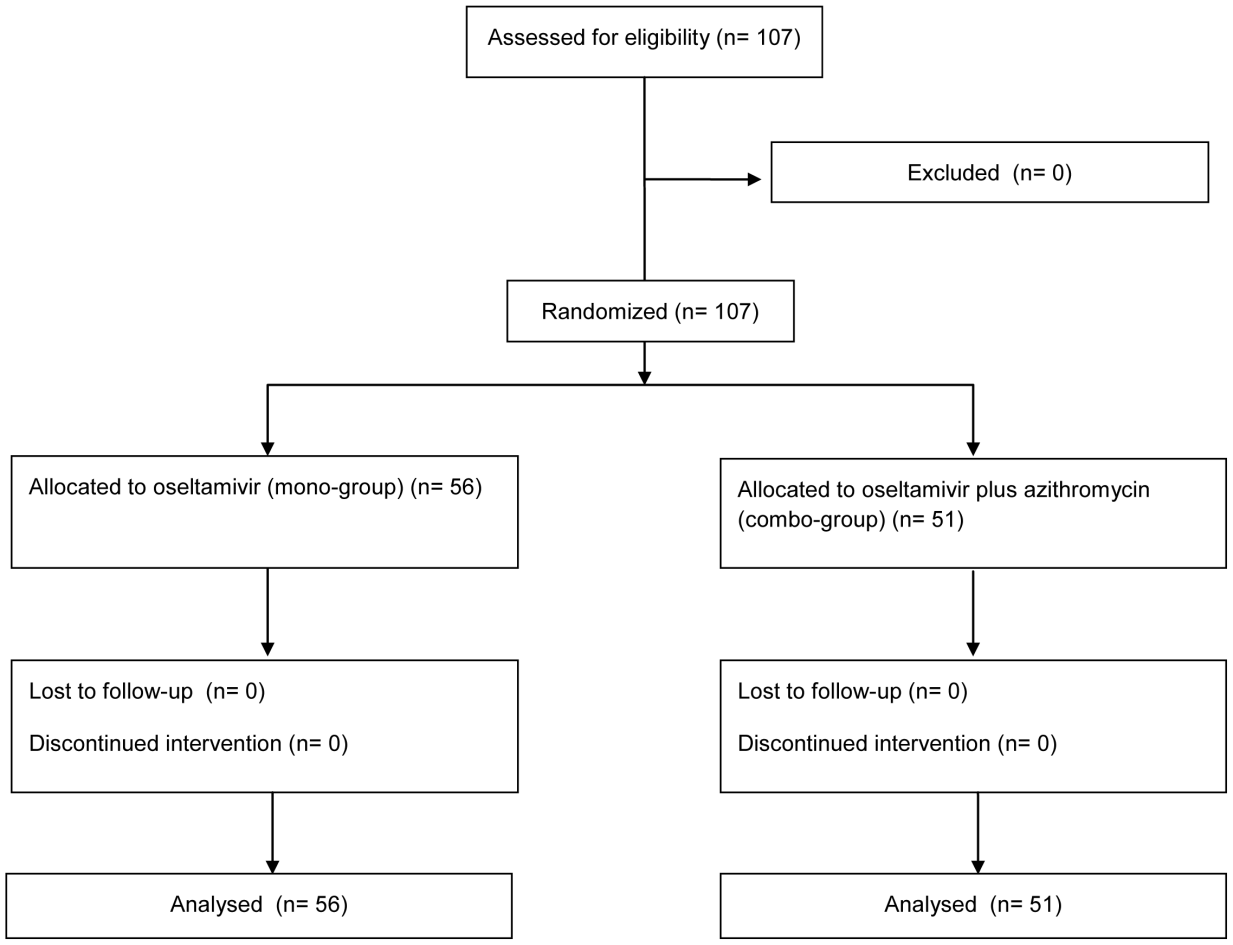
Trial Profile.

The patients were randomized into the mono-group (56 patients) or combo-group (51 patients), and all enrolled patients were included in the ITT population (Figure 1). All patients were diagnosed with influenza A virus infection, and none of the patients had comorbid pneumonia. The participants included 50 male patients and 57 female patients, and their mean age was 43.5 years. The 2 treatment groups did not differ significantly in terms of their clinical characteristics, sex, age, underlying diseases, or disease severity (Table 1).

**Table 1:** Study population in the azithromycin-oseltamivir combination therapy and oseltamivir monotherapy groups.

		Azithromycin		
		-	+	p value
No. of patients		56	51	
Age (years)	Range	20–87	20–91	0.734 (*t* test)
	Median	42	39	
	Mean ± SD	44.1±17.3	42.9±17.3	
Gender	M (%)	25 (39.3)	25 (49.0)	0.398 (Fisher)
	F (%)	31 (60.7)	26 (51.0)	
Chronic Lung Disease (%)		6 (10.7)	5 (9.8)	0.566 (Fisher)
Diabetes		3 (5.4)	0	0.140 (Fisher)
Steroid use		3(5.4)	2(3.9)	0.545(Fisher)
Maximal body temperature(mean± SD)		38.6±0.7	38.8±0.7	0.202 (*t* test)
Influenza Symptom Severity scale (ISS)				
Headache	None	8 (14.3)	7 (13.7)	0.985 (t test)
	Mild	17 (30.4)	15 (29.4)	
	Moderate	24 (42.9)	19 (37.3)	
	Severe	5 (8.9)	5 (9.8)	
Muscle/Joint pain	None	7 (7.1)	5 (9.8)	0.735 (*t* test)
	Mild	11 (19.6)	13 (25.5)	
	Moderate	24 (42.9)	19 (37.3)	
	Severe	12 (21.4)	9 (17.6)	
Heat sensation	None	3 (5.4)	2 (5.9)	0.135 (*t* test)
	Mild	11 (19.6)	6 (11.8)	
	Moderate	24 (42.9)	17 (33.3)	
	Severe	16 (28.6)	21 (41.2)	
Feeling of fatigue	None	2 (3.6)	2 (3.9)	0.738 (*t* test)
	Mild	10 (17.9)	5 (9.8)	
	Moderate	25 (44.6)	25 (49.0)	
	Severe	17 (30.4)	14 (27.5)	
Cough	None	1 (1.8)	3 (5.9)	0.014 (*t* test)
	Mild	10 (17.9)	16 (31.4)	
	Moderate	30 (53.6)	21 (41.2)	
	Severe	12 (21.4)	5 (9.8)	
Sore throat	None	11 (19.6)	10 (19.6)	0.852 (*t* test)
	Mild	26 (46.4)	19 (37.3)	
	Moderate	13 (23.2)	14 (27.5)	
	Severe	4 (7.1)	3 (5.9)	
Nasal congestion	None	16 (28.6)	11 (21.6)	0.732 (*t* test)
	Mild	12 (21.4)	17 (33.3)	
	Moderate	22 (39.3)	16 (31.4)	
	Severe	4 (7.1)	2 (3.9)	

### Inflammatory markers

**Figure 2:**
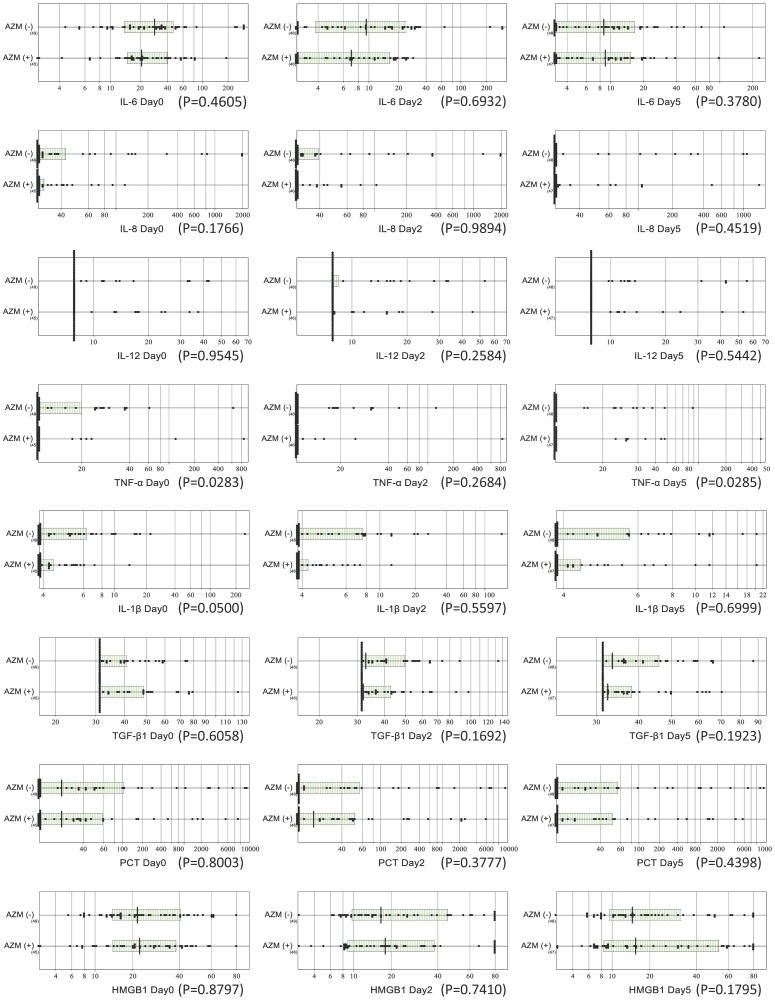
Comparison of inflammatory marker levels in the azithromycin-oseltamivir combination therapy and oseltamivir monotherapy groups. Serial test results (on days 0, 2, and 5) for the 8 inflammatory markers were compared between the groups with and without AZM. The horizontal and vertical lines depicted in the scattergram represent the central 50% range (25–75 percentiles) and the median, respectively. In the graphs for interleukin 8 (IL-8), IL-12, and tumor necrosis factor α (TNF-α), missing central boxes or concordance of the left end of the box with the median indicate that the majority of the test results were lower than the limit of detection.

The baseline values of IL-6, IL-8, IL-12, TNF-α, IL-1β, TGF-β1, PCT, and HMGB1 on day 0 before treatment allocation did not differ between the groups (Figure 2). However, the baseline values of TNF-α were statistically significantly higher in the combo-group than in the mono-group (p = 0.03). No statistically significant differences were observed between the 2 groups in the expression of any of the inflammatory cytokines or chemokines on days 2 and 5.

Although TNF-α levels were statistically different between the 2 groups on day 5, its value decreased below measurable limits in almost all patients, and thus no clear difference was observed.

### Resolution time of influenza-related symptoms

**Figure 3:**
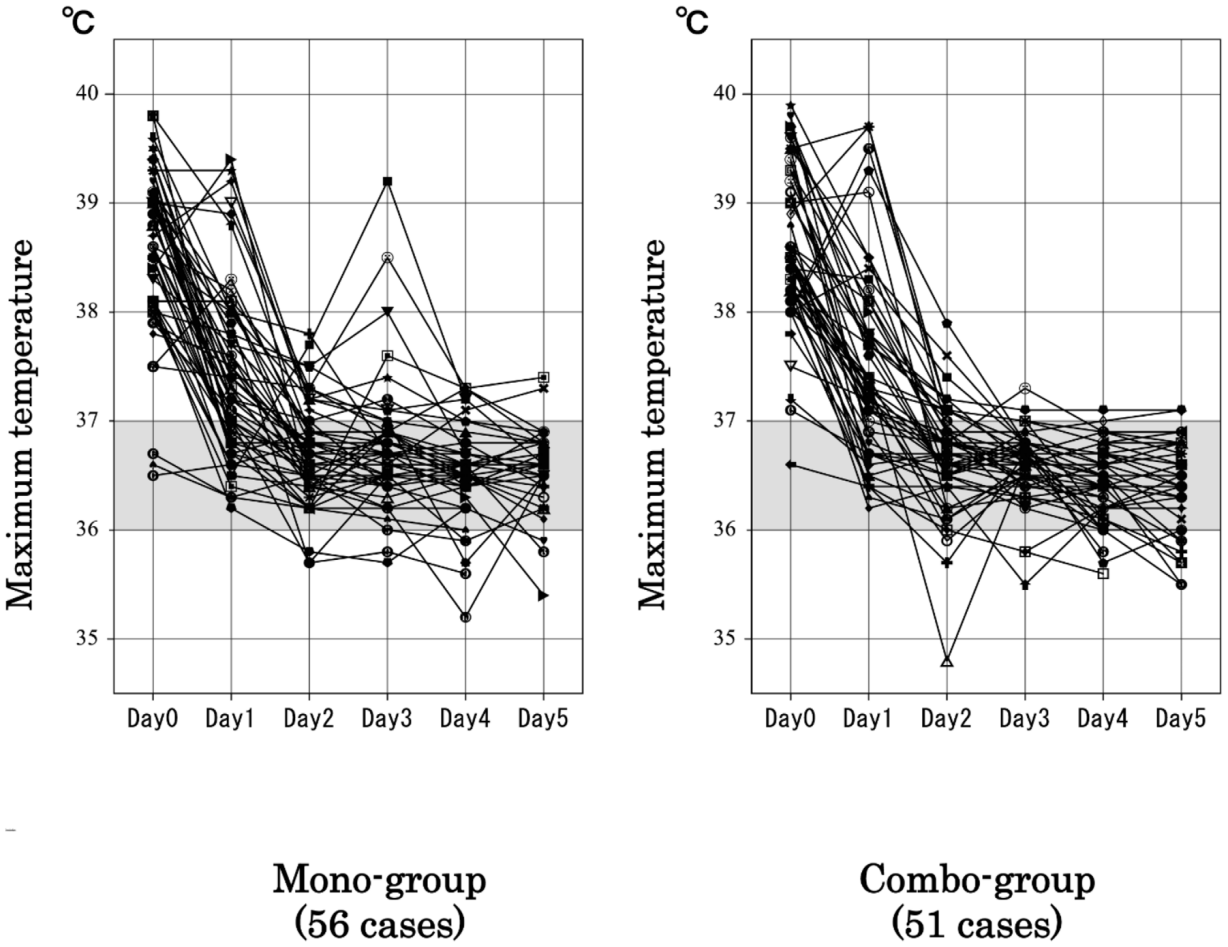
Comparisons of maximum temperature in the azithromycin-oseltamivir combination therapy and oseltamivir monotherapy groups. The maximum temperatures on day 1 through day 5 were compared between groups. No significant differences were detected between the groups on days 1, 2, 3, and 5. However, our analysis revealed a significant decrease in the maximum temperature on day 4 in the combo-group compared to that in the mono-group (p = 0.037). In addition, a mixed-design ANOVA indicated that the maximum temperature on days 3 through 5 was significantly lower in the combo-group than in the mono-group (p = 0.048).

The baseline maximal temperature on day 0 did not differ between the 2 groups (p = 0.984). Comparison of the maximum temperatures on days 1 to 5 showed no significant differences on days 1 (p = 0.864), 2 (p = 0.864), 3 (p = 0.741), or 5 (p = 0.068). However, a significant decrease in the maximum temperature was observed on day 4 between the combo-group and the mono-group (p = 0.037; Figure 3). In addition, the maximum temperature on days 3 through 5 was significantly lower in the combo-group than in the mono-group (p = 0.048).

Improvements in sore throat were observed more frequently on day 2 among patients in the combo-group than in the mono-group (p<0.05). No significant differences were observed between the 2 groups in the resolution time of other influenza-related symptoms (headache, muscle or joint pain, heat sensation, feeling of fatigue, sore throat, nasal congestion, and cough). However, compared to the mono-group, the combo-group showed a trend toward earlier resolution of fever (p = 0.05 on day 2 and p = 0.06 on day 5, Table 2).

**Table 2:** Improvement of influenza-related symptoms in the azithromycin-oseltamivir combination therapy and oseltamivir monotherapy groups.

		Azithromycin		
		−mean ± S.D	+ mean ± S.D	p value
Headache	Day0	1.48±0.86	1.48±0.89	0.9648
	ΔDay2	0.68±0.68	0.74±0.95	0.7890
	ΔDay5	1.23±0.99	1.30±0.96	0.8649
Muscle/Joint pain	Day0	1.76±0.95	1.70±0.92	0.6611
	ΔDay2	1.08±0.92	1.15±0.99	0.7968
	ΔDay5	1.60±0.93	1.6±0.95	0.9970
Heat sensation	Day0	1.98±0.86	2.24±0.85	0.1039
	ΔDay2	1.43±0.91	1.78±1.11	0.0506
	ΔDay5	1.8±0.94	2.13±1.00	0.0609
Feeling of fatigue	Day0	2.06±0.81	2.11±0.77	0.7373
	ΔDay2	0.98±0.84	1.20±1.07	0.2211
	ΔDay5	1.58±0.93	1.76±1.04	0.1871
Sore throat	Day0	1.19±0.85	1.20±0.86	0.8020
	ΔDay2	0.34±0.73	0.70±0.87	0.0323
	ΔDay5	0.81±0.83	1.02±0.88	0.2138
Nasal congestion	Day0	1.26±0.97	1.2±0.86	0.2138
	ΔDay2	0.43±1.05	0.44±0.89	0.8630
	ΔDay5	0.79±1.12	0.93±1.04	0.3890
Cough	Day0	2.00±0.71	1.62±0.78	0.0143
	ΔDay2	0.52±0.83	0.60±0.86	0.6645
	ΔDay5	1.06±0.78	1.07±0.93	0.8783

### Laboratory tests

The baseline hematological test values (hemoglobin, Ht, WBC, neutrophil count, lymphocyte count, total protein and albumin) on day 0 before treatment allocation were not significantly different between the 2 groups. Only the baseline RBC was statistically significantly higher in the combo-group than in the mono-group ((p<0.05). In addition, the combo-group showed statistically significant increases in the RBC and hemoglobin and Ht values on days 2 and 5 and a statistically significant decrease in the levels of Alb and T-P on day 2 (p<0.05 and p<0.01, respectively; Table 3).

**Table 3:** Laboratory data for the azithromycin-oseltamivir combined therapy and oseltamivir monotherapy groups.

		Azithromycin		
		- mean ± S.D (median)	+ mean ± S.D (median)	p value
WBC	Day0	4154±3442 (5045)	4137±3430 (5175)	0.7904
	ΔDay2	−1013±2091 (−1400)	−2138±1472 (−2350)	0.0103
	ΔDay5	−614±1648 (−910)	−754±2203 (−1100)	0.5667
Neutrophil	Day0	4720±1707 (4320)	4745±1524 (4540)	0.7565
	ΔDay2	−1692±2154 (−1987)	−2771±1462 (−2635)	0.0081
	ΔDay5	−1503±704 (−1286)	−1729±2143 (−1825)	0.5802
Lymphocyte	Day0	948±429 (847)	851±343 (767)	0.2722
	ΔDay2	633±540 (694)	751±346 (797)	0.1856
	ΔDay5	940±79 (918)	1170±557 (1189)	0.0614
RBC	Day0	474±46 (476)	458±40 (453)	0.0455
	ΔDay2	−3.53±20.0 (−1.36)	14.4±23.3 (13.5)	0.0002
	ΔDay5	−9.53±19.8 (−1.50)	5.9±24.1 (9.0)	0.0008
Hgb	Day0	14.3±1.47 (14.2)	13.8±1.68 (13.8)	0.1744
	ΔDay2	−0.33±0.65 (0.00)	0.43±.71 (0.50)	0.0012
	ΔDay5	−0.30±0.63 (−0.30)	0.16±0.72 (0.20)	0.0010
Hct	Day0	42.8±3.81 (42.6)	41.2±4.36 (41.4)	0.1195
	ΔDay2	−0.36±1.81 (−0.30)	1.36±2.20 (1.45)	0.0001
	ΔDay5	−1.10±1.81 (−1.30)	0.22±2.11 (0.20)	0.0010
Total Protein	Day0	7.3±0.45 (7.3)	7.2±0.44 (7.2)	0.2910
	ΔDay2	−0.22±0.33 (−0.20)	0.01±0.41 (0.05)	0.0026
	ΔDay5	−0.10±0.36 (−0.20)	0.06±0.48 (0.00)	0.0831
Alb	Day0	4.5±0.34 (4.5)	4.4±0.30 (4.5)	0.5207
	ΔDay2	−0.24±0.26 (−0.20)	−0.12±0.24 (−0.10)	0.0155
	ΔDay5	−0.20±0.22 (−0.30)	−0.02±0.29 (0.00)	0.0026
AST	Day0	24.8±9.36 (22.0)	22.6±12.38 (19.5)	0.0204
	ΔDay2	1.36±6.31 (1.0)	0.84±6.10 (2.0)	0.7559
	ΔDay5	−1.79±8.61 (−1.0)	−0.75±9.49 (0.0)	0.0609
ALT	Day0	23.3±15.02 (19.0)	24.0±21.41 (16.0)	0.4339
	ΔDay2	2.36±6.89 (1.0)	0.34±4.78 (1.0)	0.4868
	ΔDay5	1.02±7.50 (0.5)	−1.16±12.58 (0.5)	0.6777
BUN	Day0	11.3±3.60 (10.8)	11.5±4.65 (11.2)	0.9093
	ΔDay2	0.76±3.00 (1.20)	1.20±3.18 (0.80)	0.6140
	ΔDay5	0.51±3.15 (0.95)	1.57±3.11 (1.65)	0.1293
Cr	Day0	0.8±0.20 (0.8)	0.7±0.18 (0.8)	0.7492
	ΔDay2	−0.05±0.10 (−0.05)	−0.04±0.09 (−0.05)	0.5743
	ΔDay5	−0.08±0.08 (−0.06)	−0.07±0.08 (−0.06)	0.4516

### Safety

Adverse events (AEs) occurred in 11 of the 56 patients (19.6%) in the combo-group and in 9 of the 51 patients (17.6%) in the mono-group (Table 4). There was no significant difference in the incidence of AEs between the 2 groups. AEs for which a causal relationship with the study drugs could not be ruled out (known plus unknown causal relationships) occurred in 9 patients (16.1%) in the combo-group and 4 patients (7.8%) in the mono-group. No severe AE occurred in either group and no patients discontinued treatment because of an AE. The most common AEs were diarrhea (n = 3 in the combo-group) and decreased WBC (n = 5 in the combo-group and n = 3 in the mono-group). Only 1 patient in the mono-group developed secondary pneumonia.

**Table 4:** List of adverse events in the present study.

AZM	Adverse Event	Severity	Causality	Treatment
+	Secondary bronchitis	Mild	No	Continue
+	Bronchitis	Mild	No	Continue
+	Abdominal pain	Mild	Unknown	Continue
+	Abdominal pain/Diarrhea	Mild	Unknown	Continue
+	Diarrhea	Mild	Yes	Continue
+	Diarrhea	Mild	Unknown	Continue
+	Leucopenia	Mild	Unknown	Continue
+	Leucopenia	Moderate	Unknown	Continue
+	Leucopenia	Moderate	Unknown	Continue
+	Leucopenia	Mild	Unknown	Continue
+	Leucopenia	Mild	Unknown	Continue
−	Sinusitis	Moderate	No	Continue
−	Pneumonia	Mild	No	Continue
−	Bronchitis	Mild	No	Continue
−	Leucopenia	Mild	No	Continue
−	Leucopenia	Mild	Unknown	Continue
−	Leucopenia	Mild	Unknown	Continue
−	Eosinophilia	Mild	Unknown	Continue
−	Hepatic dysfunction	Mild	Unknown	Continue
−	Hepatic dysfunction	Mild	No	Continue

## Discussion

In this study, we present the findings of a randomized clinical trial of combination therapy with oseltamivir and AZM in patients with influenza virus infection. The primary endpoint of this study was variation in the expression of inflammatory markers (i.e., inflammatory cytokines and chemokines). Although the combination of oseltamivir plus AZM did not show any early reduction in the levels of inflammatory markers compared to that with oseltamivir alone, the combination treatment showed a potential early resolution of influenza-related symptoms such as fever and sore throat.

Macrolides have antibiotic and immunomodulatory activities *in vitro* and *in vivo*, and thus may have a favorable effect on the clinical outcome of patients with severe infection [6]. CAM decreases the ratio of serum IL-10 to serum TNF-α in patients with ventilator-associated pneumonia (VAP) and sepsis caused by gram-negative bacteria [16]. In addition, AZM significantly reduces the expression of the proinflammatory cytokine IL-1β and the chemokine C-C motif ligand (CCL)-2 and TNF-α in M1-induced cystic fibrosis alveolar macrophages in patients with cystic fibrosis [17]. In addition, AZM decreases acute and chronic airway inflammation in a mouse model of paramyxoviral bronchiolitis without any association with antiviral activity [18]. Azithromycin has a large volume of distribution, although serum concentrations remain low, and its half-life is much longer than that of clarithromycin. Therefore, a single dose of azithromycin is an effective and convenient dosing schedule that improves patient compliance [19]. Although we expected AZM to reduce inflammatory cytokine expression in patients with influenza virus infection, compared to oseltamivir alone, the combination of oseltamivir plus AZM did not result in an early reduction in the levels of inflammatory markers. The baseline values of each inflammatory cytokine differed for each patient, and the median value was relatively low, and therefore we suspect that variability in the patient backgrounds might have affected the study outcomes.

The present study has several potential limitations. Our study was performed during 1 winter season. Further, the number of patients was limited. We planned a sub-group analysis of older individuals, particularly patients with underlying respiratory disease. However, the number of patients who met this definition was limited, and thus the analysis was not possible. A randomized controlled trial of such patients should be performed in the future. Moreover, the enrollment criteria included patients with a wide variety of backgrounds to ensure the feasibility of patient enrollment. The timing of enrollment from the onset of an influenza-related symptom was different for each patient. Although the inclusion criteria stipulated that a patient had to be enrolled within 48 h after the onset of an influenza-related symptom, there were still 48 hours between the symptom onset and enrollment. Additionally, it was difficult to prepare a specially blinded drug for the AZM extended-release formulation because of its unique size and shape. Therefore, the study was conducted in an open-label manner. Although it cannot be denied that AZM may have had some placebo effect, we do not believe that this affected our results.

High body temperature is a common influenza-related symptom. Although no statistically significant differences in fever reduction were observed between the 2 groups until day 3, compared to oseltamivir alone, the oseltamivir plus AZM combination group showed a statistically significant early antipyretic effect on day 4. The mechanism of action of the early antipyretic effect associated with AZM is difficult to determine in our patients, but we present 2 hypotheses. The first is an anti-inflammatory effect exerted by AZM [6], and the second is a conventional antibiotic effect giving due consideration to a bacterial superinfection. In this study, we were unable to show that AZM decrease the levels of inflammatory markers in influenza patients compared to those in controls. However, macrolide therapy has been reported to improve the outcomes of patients with VAP [20] and acute lung injury (ALI) [21]. Several studies have also shown that compared to administration of beta-lactams alone, fluoroquinolones alone, or beta-lactams in combination with fluoroquinolones, administration of beta-lactams in combination with macrolides improves the survival of patients with severe community-acquired pneumonia (CAP) [22]–[24]. These reports suggest that macrolides produce an effect (i.e., anti-inflammatory or immunomodulatory) other than a conventional antibiotic effect. The potential therapeutic value of the anti-inflammatory effects of macrolides is supported by murine models of ALI induced by endotoxin [25], [26] as well as murine models of influenza [13], [27] and VAP caused by *Pseudomonas aeruginosa* [28]. These studies have shown increased survival after macrolide therapy. The possible involvement of a bacterial superinfection cannot be ruled out because bacteriological examinations such as Gram stains and cultures of respiratory samples were not required to be performed at baseline in this study. Rates of influenza-related pneumonia are generally less than 10% [29], [30], and it is very rare in Japan (1-2%) because patients tend to consult physicians at an early stage because of the broad coverage by the health insurance system. In this study, we analyzed about 100 patients, but none developed pneumonia.

The incidence of bacterial pneumonia as a secondary infection after influenza is well known as a major cause of increased morbidity and mortality. Concomitant administration of macrolides, including AZM, to treat influenza may contribute to the prevention of secondary bacterial pneumonia by preventing airway epithelial cell damage because of an overactive immune response. Macrolides exert immunomodulatory effects via inhibition of neutrophil oxidative bursts, decrease of elastase activity, and suppression of granulocyte macrophage-colony stimulating factor [6]. In this study, only 1 patient who received oseltamivir monotherapy developed secondary pneumonia, but the sample size was not sufficiently large to adequately detect secondary infection after influenza.

Decreases in serum albumin and total protein levels were significantly modulated by the addition of AZM to oseltamivir therapy. The relationship between AZM and such modulation is unclear, but AZM could have contributed to early improvement in the general condition of the patients.

In conclusion, to our knowledge, this is the first prospective, randomized, clinical trial of oseltamivir and AZM combination therapy for influenza. Although no statistically significant difference was observed in the expression levels of inflammatory cytokines and chemokines, the combination therapy showed a trend toward the earlier resolution of some symptoms.

## Supporting Information

Checklist S1 **CONSORT checklist.**
(DOC)

Protocol S1 **Trial Protocol.**
(DOC)
