# Supplementary material for: Efficacy of Combination Therapy with Oseltamivir Phosphate and Azithromycin for Influenza: A Multicenter, Open-Label, Randomized Study
Source: PLoS One. 2014 Mar 14;9(3):e91293. doi: 10.1371/journal.pone.0091293 (PMC3954629; doi:10.1371/journal.pone.0091293)
Supplement: Protocol S1 — Trial Protocol. (DOC) [file pone.0091293.s002.doc]

Protocol No. NEOCI-0007

**臨床試験実施計画書**

「インフルエンザによる炎症および組織障害に対するオセルタミビルリン酸塩（タミフル®）とアジスロマイシン単回投与製剤

（ジスロマック®SR成人用ドライシロップ2g）併用の有効性検討」

版番号：第1版　2010年 9月 6日 作成

第2版　2010年10月29日　作成

**１．緒言**

2009年の新型インフルエンザ（Swine Flu）では発熱から2-3日で肺炎や脳症へ重篤化する症例が散見されており、基礎疾患を有する患者では特に肺炎へ重篤化するリスクが高い可能性が示唆されている。インフルエンザにおける肺炎は、細菌による混合感染や二次性肺炎のほか、インフルエンザウイルスそのものによる肺炎があり、インフルエンザにおける肺炎では、炎症性サイトカインや細胞障害因子である**high mobility group B1(HMGB1)**の関与が示唆されている。更に、将来流行が懸念される高病原性鳥インフルエンザでは、多くの症例が臨床的には重症肺炎になることが予想され、これまでの解析から、その原因としてサイトカインストームが関与していると考えられている。サイトカインストームによって重篤となる場合には、血液浄化などの抗サイトカイン療法も考慮されるが、厳重な管理が必要な治療法であり、ガイドラインでは「高次医療施設で行うことが望ましい」とされ、プライマリーケアでは対応が難しい。

新型インフルエンザの蔓延により重篤化する患者が増えると、医療体制の崩壊にもつながる可能性があり、プライマリーケアで重篤化を抑えられる治療法は患者のみならず医療体制を堅持するためにも有用である。

アジスロマイシン単回投与製剤は、抗菌作用以外にも各種サイトカインに対する抑制作用を有することで、二次的肺炎に対して有効であることが基礎試験において認められている。そこで、今回、抗インフルエンザ薬であるオセルタミビルリン酸塩単独投与群と比較してアジスロマイシン単回投与製剤併用群がインフルエンザによる炎症及び組織障害に関する因子に対して有効であることを検討する。

**２．目的**

　　インフルエンザによる炎症及び組織障害に関する因子に対するオセルタミビルリン酸塩単独とアジスロマイシン単回投与製剤併用の有効性を検討する。

**３．実施方法**

3.1.試験薬（薬剤名）

1）オセルタミビルリン酸塩（タミフル®）

2）アジスロマイシン単回投与製剤（ジスロマック®SR成人用ドライシロップ2g）

3.2.対象

1）Swine flu もしくは季節性インフルエンザに罹患し、選択基準を満たす患者。

3.3.インフルエンザ症状重症度評価項目（ISS）※1

1）全身症状

頭痛、筋肉又は関節の痛み、熱っぽさ又は悪寒、疲労感

2）呼吸器症状

咳、喉の痛み、鼻づまり

上記症状を以下の4段階で評価する。

0：なし（通常の状態）

1：軽症（ほとんど気にならない）

2：中程度（かなり気になる）

3：重症（我慢できない）

3.4.選択基準

下記1）～6)の条件を満たすインフルエンザ様症状を呈する患者

1）年齢20歳以上

2）投与前に試験参加を文書により同意した患者

3）発熱（38℃以上）により外来受診した患者

4）インフルエンザ症状重症度評価項目（ISS）※1のうち、中程度以上の症状を

2項目以上有する患者

5）発症から48時間以内の患者（発症時間の定義は以下のいずれかとする）

①体温が初めて上昇した時（患者の平熱から、1℃以上上昇）

②インフルエンザ症状重症度評価項目（ISS）症状を１項目以上経験した時

6）迅速診断キットで陽性となった患者

3.5.除外基準

1）試験薬に対して過敏症を有する患者

2）試験薬との併用禁忌な薬を服用している患者

3）医師が不適当と判断した場合（臨床的に明らかな細菌感染の患者等）

4）マクロライド少量長期投与中の患者

3.6.試験の方法

3.6.1.被験者の同意

選択基準を満たし、除外基準に抵触しない患者に対し、同意を取得する。

3.6.2.症例登録及び薬剤割付

　　　中央登録方式を用い症例登録を行う。また、ハイリスク因子の有無を割付因子とし、最小化法にてオセルタミビルリン酸塩単独群（A群）またはアジスロマイシン単回投与製剤併用群（B群）の2群に薬剤を割り付ける。

　3.6.3.投与方法

1）A群：オセルタミビルリン酸塩単独群
タミフル®カプセル1回１カプセル（75mg）を1日2回、5日間内服。

　　　2）B群：アジスロマイシン単回投与製剤併用群

　　　　　タミフル®カプセル1回１カプセル（75mg）を1日2回、5日間内服
投与開始日のタミフル®内服後の空腹時にジスロマック®SRドライシロップ2g

を内服。ジスロマック®SRドライシロップ2gは、添付文書に従い、食後2 時間以上の空腹時に服用する。服用後は、次の食事を2 時間以上控えること。なお、ジスロマック®SRドライシロップ2gの副作用として下痢等が認められているため、必要に応じてセレキノン®の併用を可とする。

用法用量は各添付文書を遵守する。

　3.6.3.観察・検査項目

| 観察･検査  調査項目 | 投与  開始前 | 投与  1日後 | 投与  2日後 | 投与  3日後 | 投与  4日後 | 投与  5日後 | 投与  6日後 | 投与  7日後 |
| --- | --- | --- | --- | --- | --- | --- | --- | --- |
| 許容範囲 | -1～0 |  | 2日後+1日 | |  | 5日後+2日 | | |
| 同意取得 | ● |  |  |  |  |  |  |  |
| 診察 | ● |  | ● |  |  | ● |  |  |
| 患者背景 | ● |  |  |  |  |  |  |  |
| ウイルス学的検査 | ● |  |  |  |  |  |  |  |
| ﾊﾞｲﾀﾙｻｲﾝ | ● |  | ● |  |  | ● |  |  |
| インフルエンザ症状  重症度評価  項目（ISS） | ● |  | 症状日誌に患者自身で記入 |  |  |  |  |  |
| 臨床検査 | ● |  | ● |  |  | ● |  |  |
| 胸部X線 | ● |  |  |  |  |  |  |  |
| ｻｲﾄｶｲﾝ検査 | ● |  | ● |  |  | ● |  |  |
| 有害事象 |  |  |  |  |  |  |  |  |
| 症状日誌 | 提供 |  |  |  |  | 回収 |  |  |

　　1）患者背景

年齢、性別、体重、基礎疾患（呼吸器、心、肝、腎、内分泌代謝疾患）の有無、妊娠の有無、併用薬剤の有無とその内容、インフルエンザワクチン接種歴の有無

　 2）ハイリスク因子

　　　下記のハイリスク因子について確認し、調査票へ記載する。

　　　　・年齢65歳以上

　　　　・コントロール不良の糖尿病（HbA1c＊：7.0以上）

＊試験への登録前、4週間以内のHbA1c値で判断

・薬物治療中の慢性呼吸器疾患（例：COPD，気管支喘息）

・免疫機能を抑制する次の薬剤を使用中：経口又は吸入による副腎皮質ステロイド薬（プレドニゾロン換算10mg/日以上）、免疫抑制剤（タクロリムスを除く）

　3）ウイルス学的検査：迅速診断

　　4）バイタルサイン：体温、脈拍数、血圧

5）インフルエンザ症状重症度評価項目（ISS）3.3.※1参照

　　投与開始前のみ医師の問診にて確認し、その後は症状日誌に患者自身が記録する。

　6）臨床検査

①血液学：赤血球数・Hb・Ht・血小板数・白血球数・白血球数分画

②生化学：AST・ALT・T-Bil・BUN・Cre・T-P・Alb・Na・Cl・K

③免疫学：CRP

　　7）胸部X線撮影

　　8）サイトカイン検査

　　　　炎症性サイトカイン・ケモカイン(TNF-α, IL-1β,6,8,12, TGF-β)、PCT、HMGB1

　　9）有害事象

　　　　試験薬が投与された際に起こる、あらゆる好ましくないあるいは意図しない徴候（臨床検査値の異常を含む）、症状または病気のことであり、当該医薬品との因果関係の有無は問わない。なお、インフルエンザ症状の悪化は、有害事象とはせず有効性に関する評価項目として評価する。肺炎・気管支炎・中耳炎・副鼻腔炎については、インフルエンザ関連合併症として調査票に記載する。

3.5.4.症状日誌

　　1）投与開始日に被験者へ症状日誌を提供し、記録方法を指導する。

　　2）記録内容：全ての項目について夕方（18:00頃）に記録する。

　　　　①最高体温

　　　　　当日の最高体温を記録する。

　　　　②インフルエンザ症状重症度評価項目（ISS）3.3.※1参照

　　　　③活動評価（IIWS）

　　　　　通常活動を行う能力についてVASを用い、記録する。

　　3）投与5日後来院時に患者日誌を持参するよう指導し、記載内容を確認したうえで

回収する。

3.5.5.サイトカイン検査の搬送方法

　1）真空採血管に採血後、遠心分離機を用いて血清を分離した状態で施設冷凍庫にて凍結。翌日以降凍結した状態で試験事務局へ搬送。凍結状態が確保できていれば搬送方法は問わない。

**４．評価項目**

　4.1.主要評価項目

1）炎症性サイトカイン・ケモカイン、HMGB1、PCTの変動

　4.2.副次的評価項目

1）インフルエンザ罹患期間（診断時点からインフルエンザ症状が消失するまでの時間）

2）インフルエンザ関連合併症（肺炎、気管支炎、中耳炎、副鼻腔炎）の発現率

3）各インフルエンザ症状が消失するまでの時間

4）有害事象及び副作用

**５．統計解析**

本試験の主目的は探索的であることから、各評価項目におけるデータの記述ならびに関心のあるパラメータの推定に主眼をおき、統計的仮説検定は参考的位置づけとする。なお、信頼区間の信頼係数は0.95、仮設検定の有意水準は0.05（両側）とする。また、解析対象集団について、無作為化され試験薬が投与された全例をIntention to Treat集団（ITT集団）と定義する。

主要評価項目である炎症性サイトカイン関連の検査項目については、ITT集団のうち投与開始日、投与２日後および投与５日後の検査値を有する症例を対象に実施する。各検査項目について適切な記述統計を用いて要約する。また参考として、各検査項目について投与前後の差を症例毎に算出し、これらの群間比較についてWilcoxon順位和検定を適用する。

インフルエンザ罹患期間については、ITT集団を対象に、Kaplan-Meier法により各投与群の生存時間曲線および罹患期間の中央値を推定する。また、Coxの比例ハザードモデルを用いてハザード比を推定する。なお、最終観察日までにインフルエンザ症状が消失しなかった症例は、解析上「脱落」として取り扱う。また参考として、罹患期間の群間比較についてLog-rank検定を適用する。

インフルエンザ関連合併症の各症状については7日間内に発現した症例の発現割合を推定する。解析対象はITT集団とする。また参考として、各症状の発現割合の群間比較についてFisherの直接法を用いて検定する。

各インフルエンザ症状が消失するまでの時間については、ITT集団を対象に実施する。

解析方法は上記のインフルエンザ罹患期間と同様とする。上記に加えて、評価項目間

の関連性などについて、必要に応じて探索的データ解析を実施する。

オセルタミビルリン酸塩単独投与におけるインフルエンザ罹患期間の中央値は81.8時間（95%信頼区間、73.2，91.1）と報告されている^18)^。アジスロマイシン単回投与製剤の併用によりオセルタミビルリン酸塩単独投与と比べインフルエンザ罹患期間の中央値が20％短縮すると仮定し、１群50例（２群計100例）で試験を行った場合、アジスロマイシン単回投与製剤併用群の罹患期間の中央値の点推定値が、オセルタミビルリン酸塩単独群のそれよりも小さい値をとる確率は約78%^※2^となる。また、インフルエンザ症状が消失するまでの時間において、オセルタミビルリン酸塩単独投与群のアジスロマイシン単回投与製剤併用群に対するハザード比の点推定値が１未満（アジスロマイシン単回投与製剤併用群に有利）になる確率は約85%^※2^となる。なお、２群間の差の検定（両側、有意水準5%）の検出力は約17％となる。(^※2^指数分布に基づくシミュレーションによる)

また、各インフルエンザ関連合併症の発現率がオセルタミビルリン酸塩単独群で10%, 15%, 20%、アジスロマイシン単回投与製剤併用群で1%、5%と仮定し、１群50例（2群計100例）で試験を行った場合、Fisherの直接法（両側、有意水準５％）による発現率の群間比較の検出力は下表の通りとなる。

| アジスロマイシン単回投与製剤併用群での発現率 | オセルタミビルリン酸塩単独群での発現率 | | |
| --- | --- | --- | --- |
|  | 10% | 15% | 20% |
| 1% | 27% | 64% | 87% |
| 5% | 6% | 24% | 51% |

**６．実施期間および収集予定例数**

6.1.実施期間

2010年10月～2011年9月

　6.2.収集予定例数

　　A群（オセルタミビルリン酸塩単独群）：50例

B群（アジスロマイシン単回投与製剤併用群）：50例　　　計100例

**７．代表世話人**

　　長崎大学病院　病院長　河野 茂

**８．主任研究者**

　　長崎大学病院　第二内科　関　雅文

〒852-8501　長崎県 長崎市 坂本1丁目 7番1号

TEL:(095)819-7273 FAX:(095)849-7285

E-mail: seki@nagasaki-u.ac.jp

**９．試験事務局**

特定非営利活動法人NEOCI　　(Nagasaki evaluation organization for clinical interventions)

掛屋　弘

〒852-8117　長崎県長崎市平野町11-13宮村ビル2階

TEL:095-842-5402　FAX:095-842-5401

E-Mail :shinkin@peath.co.jp

**１０．参加予定施設（１４施設）**

1）長崎大学病院

2）日本赤十字社長崎原爆諌早病院

3）日本赤十字社長崎原爆病院

4）北松中央病院

5）佐世保市立総合病院

6）国立病院機構　嬉野医療センター

7）医療法人 光晴会病院

8）健康保険諫早総合病院

9）長崎市立市民病院

10）おにつか内科・消化器科

11）医療法人　はやしだ内科

12）ともなが内科クリニック

13）入船クリニック

14）かわむら内科

**１１．倫理的事項**

1）遵守事項

本試験は「ヘルシンキ宣言」の精神及び「臨床研究に関する倫理指針」を遵守して実施する。

2）倫理審査委員会

本試験実施に先立ち、各医療機関にて倫理（審査）委員会において本試験の科学性及び倫理性を検討する。本試験実施計画書は各医療機関の試験責任医師より医療機関長を通じて倫理（審査）委員会へ提出され、承認を得なければならない。但し、医療機関が小規模であること等により当該医療機関内に倫理審査委員会を設置できない場合には、長崎大学病院倫理審査委員会での承認をもってこれに代えることができる。

3）インフォームド・コンセント

試験担当医師は被験者が試験に参加する前に被験者（又は代諾者）に対して同意文書及び説明文書を用いて十分に説明し、試験への参加について自由意思による同意を文書により得るものとする。

4）個人情報保護法の遵守

成果の公表に関しては、個人情報保護法に基づいて被験者の名前や住所などプライバシーにかかわる事項や個人を特定できるような試験結果は一切公表しない。また、個人情報の取扱いを第三者に委託する場合は、個人情報を適切に管理できる者を委託先として選定し、委託先との間で個人情報保護のために必要な事項を取り決め、また、個人情報の取扱いについて適正に監督・管理することにより、個人情報保護を十分に図ることとする。

5）健康被害に関する補償

本試験に登録される被験者はインフルエンザと診断され、オセルタミビルリン酸塩単独群若しくはアジスロマイシン単回投与製剤併用群として、保険診療の範囲で投与される。なお、プロトコールに起因する健康被害については、試験事務局にて加入している賠償責任保険にて対応する。しかしながら、重篤な合併症の存在やインフルエンザに対する治療法の特性から死亡を含む重篤な健康被害が発生する可能性は否定できないため、各医療機関の試験責任医師及び担当医師は、本試験中に健康被害による賠償責任が生じた場合の履行措置として医師賠償責任保険に加入する。

**１２．参考資料・文献リスト**

1. [Seki M](http://www.ncbi.nlm.nih.gov/sites/entrez?Db=pubmed&Cmd=Search&Term="Seki M"%5BAuthor%5D&itool=EntrezSystem2.PEntrez.Pubmed.Pubmed_ResultsPanel.Pubmed_DiscoveryPanel.Pubmed_RVAbstractPlus), [Kohno](http://www.ncbi.nlm.nih.gov/sites/entrez?Db=pubmed&Cmd=Search&Term="Kosai K"%5BAuthor%5D&itool=EntrezSystem2.PEntrez.Pubmed.Pubmed_ResultsPanel.Pubmed_DiscoveryPanel.Pubmed_RVAbstractPlus) S, Newstead M, Zeng X, Bhan U, Lukacs N, Kunkel S, Standiford T. Critical role of IRAK-M in regulating chemokine-dependent deleterious inflammation in murine influenza pneumonia. *J Immunol* 2010,184,1410-8.
2. [Seki M](http://www.ncbi.nlm.nih.gov/sites/entrez?Db=pubmed&Cmd=Search&Term="Seki M"%5BAuthor%5D&itool=EntrezSystem2.PEntrez.Pubmed.Pubmed_ResultsPanel.Pubmed_DiscoveryPanel.Pubmed_RVAbstractPlus), [Kosai K](http://www.ncbi.nlm.nih.gov/sites/entrez?Db=pubmed&Cmd=Search&Term="Kosai K"%5BAuthor%5D&itool=EntrezSystem2.PEntrez.Pubmed.Pubmed_ResultsPanel.Pubmed_DiscoveryPanel.Pubmed_RVAbstractPlus), Hara A, [Imamura Y](http://www.ncbi.nlm.nih.gov/sites/entrez?Db=pubmed&Cmd=Search&Term="Imamura Y"%5BAuthor%5D&itool=EntrezSystem2.PEntrez.Pubmed.Pubmed_ResultsPanel.Pubmed_DiscoveryPanel.Pubmed_RVAbstractPlus), [Nakamura S](http://www.ncbi.nlm.nih.gov/sites/entrez?Db=pubmed&Cmd=Search&Term="Nakamura S"%5BAuthor%5D&itool=EntrezSystem2.PEntrez.Pubmed.Pubmed_ResultsPanel.Pubmed_DiscoveryPanel.Pubmed_RVAbstractPlus), [Kurihara S](http://www.ncbi.nlm.nih.gov/sites/entrez?Db=pubmed&Cmd=Search&Term="Kurihara S"%5BAuthor%5D&itool=EntrezSystem2.PEntrez.Pubmed.Pubmed_ResultsPanel.Pubmed_DiscoveryPanel.Pubmed_RVAbstractPlus), [Izumikawa K](http://www.ncbi.nlm.nih.gov/sites/entrez?Db=pubmed&Cmd=Search&Term="Izumikawa K"%5BAuthor%5D&itool=EntrezSystem2.PEntrez.Pubmed.Pubmed_ResultsPanel.Pubmed_DiscoveryPanel.Pubmed_RVAbstractPlus), [Kakeya H](http://www.ncbi.nlm.nih.gov/sites/entrez?Db=pubmed&Cmd=Search&Term="Kakeya H"%5BAuthor%5D&itool=EntrezSystem2.PEntrez.Pubmed.Pubmed_ResultsPanel.Pubmed_DiscoveryPanel.Pubmed_RVAbstractPlus), [Yamamoto Y](http://www.ncbi.nlm.nih.gov/sites/entrez?Db=pubmed&Cmd=Search&Term="Yamamoto Y"%5BAuthor%5D&itool=EntrezSystem2.PEntrez.Pubmed.Pubmed_ResultsPanel.Pubmed_DiscoveryPanel.Pubmed_RVAbstractPlus), [Yanagihara K](http://www.ncbi.nlm.nih.gov/sites/entrez?Db=pubmed&Cmd=Search&Term="Yanagihara K"%5BAuthor%5D&itool=EntrezSystem2.PEntrez.Pubmed.Pubmed_ResultsPanel.Pubmed_DiscoveryPanel.Pubmed_RVAbstractPlus), Miyazaki Y, Mukae H, [Tashiro T](http://www.ncbi.nlm.nih.gov/sites/entrez?Db=pubmed&Cmd=Search&Term="Tashiro T"%5BAuthor%5D&itool=EntrezSystem2.PEntrez.Pubmed.Pubmed_ResultsPanel.Pubmed_DiscoveryPanel.Pubmed_RVAbstractPlus), [Kohno S](http://www.ncbi.nlm.nih.gov/sites/entrez?Db=pubmed&Cmd=Search&Term="Kohno S"%5BAuthor%5D&itool=EntrezSystem2.PEntrez.Pubmed.Pubmed_ResultsPanel.Pubmed_DiscoveryPanel.Pubmed_RVAbstractPlus). Expression and analysis of Platelet activating factor (PAF) related molecule in severe pneumonia in mice due to influenza virus and bacterial co-infection using by DNA microarray [*Jpn J Infect Dis.*](javascript:AL_get(this, 'jour', 'Jpn J Infect Dis.');)2009. 62; 6-10.
3. Yanagihara K, Izumikawa K, Higa F, Tateyama M, Tokimatsu I, Hiramatsu K, Fujita J, Kadota J, Kohno S. [Efficacy of azithromycin in the treatment of community-acquired pneumonia, including patients with macrolide-resistant Streptococcus pneumoniae infection.](http://www.ncbi.nlm.nih.gov/pubmed/19336954?ordinalpos=2&itool=EntrezSystem2.PEntrez.Pubmed.Pubmed_ResultsPanel.Pubmed_DefaultReportPanel.Pubmed_RVDocSum) *Intern Med.* 2009; 48: 527-35.
4. [Karlström A](http://www.ncbi.nlm.nih.gov/sites/entrez?Db=pubmed&Cmd=Search&Term="Karlström A"%5BAuthor%5D&itool=EntrezSystem2.PEntrez.Pubmed.Pubmed_ResultsPanel.Pubmed_DiscoveryPanel.Pubmed_RVAbstractPlus), [Boyd KL](http://www.ncbi.nlm.nih.gov/sites/entrez?Db=pubmed&Cmd=Search&Term="Boyd KL"%5BAuthor%5D&itool=EntrezSystem2.PEntrez.Pubmed.Pubmed_ResultsPanel.Pubmed_DiscoveryPanel.Pubmed_RVAbstractPlus), [English BK](http://www.ncbi.nlm.nih.gov/sites/entrez?Db=pubmed&Cmd=Search&Term="English BK"%5BAuthor%5D&itool=EntrezSystem2.PEntrez.Pubmed.Pubmed_ResultsPanel.Pubmed_DiscoveryPanel.Pubmed_RVAbstractPlus), [McCullers JA](http://www.ncbi.nlm.nih.gov/sites/entrez?Db=pubmed&Cmd=Search&Term="McCullers JA"%5BAuthor%5D&itool=EntrezSystem2.PEntrez.Pubmed.Pubmed_ResultsPanel.Pubmed_DiscoveryPanel.Pubmed_RVAbstractPlus). Treatment with protein synthesis inhibitors improves outcomes of secondary bacterial pneumonia after influenza. [*J Infect Dis*.](javascript:AL_get(this, 'jour', 'J Infect Dis.');) 2009; 199: 311-9.
5. [Seki M](http://www.ncbi.nlm.nih.gov/sites/entrez?Db=pubmed&Cmd=Search&Term="Seki M"%5BAuthor%5D&itool=EntrezSystem2.PEntrez.Pubmed.Pubmed_ResultsPanel.Pubmed_DiscoveryPanel.Pubmed_RVAbstractPlus), Suyama N, Hashiguchi K, Hara A, [Kosai K](http://www.ncbi.nlm.nih.gov/sites/entrez?Db=pubmed&Cmd=Search&Term="Kosai K"%5BAuthor%5D&itool=EntrezSystem2.PEntrez.Pubmed.Pubmed_ResultsPanel.Pubmed_DiscoveryPanel.Pubmed_RVAbstractPlus), [Yanagihara K](http://www.ncbi.nlm.nih.gov/sites/entrez?Db=pubmed&Cmd=Search&Term="Yanagihara K"%5BAuthor%5D&itool=EntrezSystem2.PEntrez.Pubmed.Pubmed_ResultsPanel.Pubmed_DiscoveryPanel.Pubmed_RVAbstractPlus), [Nakamura S](http://www.ncbi.nlm.nih.gov/sites/entrez?Db=pubmed&Cmd=Search&Term="Nakamura S"%5BAuthor%5D&itool=EntrezSystem2.PEntrez.Pubmed.Pubmed_ResultsPanel.Pubmed_DiscoveryPanel.Pubmed_RVAbstractPlus), [Kurihara S](http://www.ncbi.nlm.nih.gov/sites/entrez?Db=pubmed&Cmd=Search&Term="Kurihara S"%5BAuthor%5D&itool=EntrezSystem2.PEntrez.Pubmed.Pubmed_ResultsPanel.Pubmed_DiscoveryPanel.Pubmed_RVAbstractPlus), [Imamura Y](http://www.ncbi.nlm.nih.gov/sites/entrez?Db=pubmed&Cmd=Search&Term="Imamura Y"%5BAuthor%5D&itool=EntrezSystem2.PEntrez.Pubmed.Pubmed_ResultsPanel.Pubmed_DiscoveryPanel.Pubmed_RVAbstractPlus), [Izumikawa K](http://www.ncbi.nlm.nih.gov/sites/entrez?Db=pubmed&Cmd=Search&Term="Izumikawa K"%5BAuthor%5D&itool=EntrezSystem2.PEntrez.Pubmed.Pubmed_ResultsPanel.Pubmed_DiscoveryPanel.Pubmed_RVAbstractPlus), [Kakeya H](http://www.ncbi.nlm.nih.gov/sites/entrez?Db=pubmed&Cmd=Search&Term="Kakeya H"%5BAuthor%5D&itool=EntrezSystem2.PEntrez.Pubmed.Pubmed_ResultsPanel.Pubmed_DiscoveryPanel.Pubmed_RVAbstractPlus), [Yamamoto Y](http://www.ncbi.nlm.nih.gov/sites/entrez?Db=pubmed&Cmd=Search&Term="Yamamoto Y"%5BAuthor%5D&itool=EntrezSystem2.PEntrez.Pubmed.Pubmed_ResultsPanel.Pubmed_DiscoveryPanel.Pubmed_RVAbstractPlus), Mukae H, [Tashiro T](http://www.ncbi.nlm.nih.gov/sites/entrez?Db=pubmed&Cmd=Search&Term="Tashiro T"%5BAuthor%5D&itool=EntrezSystem2.PEntrez.Pubmed.Pubmed_ResultsPanel.Pubmed_DiscoveryPanel.Pubmed_RVAbstractPlus), [Kohno S](http://www.ncbi.nlm.nih.gov/sites/entrez?Db=pubmed&Cmd=Search&Term="Kohno S"%5BAuthor%5D&itool=EntrezSystem2.PEntrez.Pubmed.Pubmed_ResultsPanel.Pubmed_DiscoveryPanel.Pubmed_RVAbstractPlus). A patient with fulminant influenza-related bacterial pneumonia due to *Streptococcus pneumoniae* followed by *Mycobacterium tuberculosis* infection *Intern Med* 2008; 47: 2043-2047.
6. [Kosai K](http://www.ncbi.nlm.nih.gov/sites/entrez?Db=pubmed&Cmd=Search&Term="Kosai K"%5BAuthor%5D&itool=EntrezSystem2.PEntrez.Pubmed.Pubmed_ResultsPanel.Pubmed_DiscoveryPanel.Pubmed_RVAbstractPlus), [Seki M](http://www.ncbi.nlm.nih.gov/sites/entrez?Db=pubmed&Cmd=Search&Term="Seki M"%5BAuthor%5D&itool=EntrezSystem2.PEntrez.Pubmed.Pubmed_ResultsPanel.Pubmed_DiscoveryPanel.Pubmed_RVAbstractPlus), [Yanagihara K](http://www.ncbi.nlm.nih.gov/sites/entrez?Db=pubmed&Cmd=Search&Term="Yanagihara K"%5BAuthor%5D&itool=EntrezSystem2.PEntrez.Pubmed.Pubmed_ResultsPanel.Pubmed_DiscoveryPanel.Pubmed_RVAbstractPlus), [Nakamura S](http://www.ncbi.nlm.nih.gov/sites/entrez?Db=pubmed&Cmd=Search&Term="Nakamura S"%5BAuthor%5D&itool=EntrezSystem2.PEntrez.Pubmed.Pubmed_ResultsPanel.Pubmed_DiscoveryPanel.Pubmed_RVAbstractPlus), [Kurihara S](http://www.ncbi.nlm.nih.gov/sites/entrez?Db=pubmed&Cmd=Search&Term="Kurihara S"%5BAuthor%5D&itool=EntrezSystem2.PEntrez.Pubmed.Pubmed_ResultsPanel.Pubmed_DiscoveryPanel.Pubmed_RVAbstractPlus), [Imamura Y](http://www.ncbi.nlm.nih.gov/sites/entrez?Db=pubmed&Cmd=Search&Term="Imamura Y"%5BAuthor%5D&itool=EntrezSystem2.PEntrez.Pubmed.Pubmed_ResultsPanel.Pubmed_DiscoveryPanel.Pubmed_RVAbstractPlus), [Izumikawa K](http://www.ncbi.nlm.nih.gov/sites/entrez?Db=pubmed&Cmd=Search&Term="Izumikawa K"%5BAuthor%5D&itool=EntrezSystem2.PEntrez.Pubmed.Pubmed_ResultsPanel.Pubmed_DiscoveryPanel.Pubmed_RVAbstractPlus), [Kakeya H](http://www.ncbi.nlm.nih.gov/sites/entrez?Db=pubmed&Cmd=Search&Term="Kakeya H"%5BAuthor%5D&itool=EntrezSystem2.PEntrez.Pubmed.Pubmed_ResultsPanel.Pubmed_DiscoveryPanel.Pubmed_RVAbstractPlus), [Yamamoto Y](http://www.ncbi.nlm.nih.gov/sites/entrez?Db=pubmed&Cmd=Search&Term="Yamamoto Y"%5BAuthor%5D&itool=EntrezSystem2.PEntrez.Pubmed.Pubmed_ResultsPanel.Pubmed_DiscoveryPanel.Pubmed_RVAbstractPlus), [Tashiro T](http://www.ncbi.nlm.nih.gov/sites/entrez?Db=pubmed&Cmd=Search&Term="Tashiro T"%5BAuthor%5D&itool=EntrezSystem2.PEntrez.Pubmed.Pubmed_ResultsPanel.Pubmed_DiscoveryPanel.Pubmed_RVAbstractPlus), [Kohno S](http://www.ncbi.nlm.nih.gov/sites/entrez?Db=pubmed&Cmd=Search&Term="Kohno S"%5BAuthor%5D&itool=EntrezSystem2.PEntrez.Pubmed.Pubmed_ResultsPanel.Pubmed_DiscoveryPanel.Pubmed_RVAbstractPlus). Two-dimensional gel electrophoresis analysis in simultaneous influenza pneumonia and bacterial infection in mice. [*Clin Exp Immunol.*](javascript:AL_get(this, 'jour', 'Clin Exp Immunol.');) 2008, 152: 364-371.
7. [Kosai K](http://www.ncbi.nlm.nih.gov/sites/entrez?Db=pubmed&Cmd=Search&Term="Kosai K"%5BAuthor%5D&itool=EntrezSystem2.PEntrez.Pubmed.Pubmed_ResultsPanel.Pubmed_DiscoveryPanel.Pubmed_RVAbstractPlus), [Seki M](http://www.ncbi.nlm.nih.gov/sites/entrez?Db=pubmed&Cmd=Search&Term="Seki M"%5BAuthor%5D&itool=EntrezSystem2.PEntrez.Pubmed.Pubmed_ResultsPanel.Pubmed_DiscoveryPanel.Pubmed_RVAbstractPlus), [Yanagihara K](http://www.ncbi.nlm.nih.gov/sites/entrez?Db=pubmed&Cmd=Search&Term="Yanagihara K"%5BAuthor%5D&itool=EntrezSystem2.PEntrez.Pubmed.Pubmed_ResultsPanel.Pubmed_DiscoveryPanel.Pubmed_RVAbstractPlus), [Nakamura S](http://www.ncbi.nlm.nih.gov/sites/entrez?Db=pubmed&Cmd=Search&Term="Nakamura S"%5BAuthor%5D&itool=EntrezSystem2.PEntrez.Pubmed.Pubmed_ResultsPanel.Pubmed_DiscoveryPanel.Pubmed_RVAbstractPlus), [Kurihara S](http://www.ncbi.nlm.nih.gov/sites/entrez?Db=pubmed&Cmd=Search&Term="Kurihara S"%5BAuthor%5D&itool=EntrezSystem2.PEntrez.Pubmed.Pubmed_ResultsPanel.Pubmed_DiscoveryPanel.Pubmed_RVAbstractPlus), [Izumikawa K](http://www.ncbi.nlm.nih.gov/sites/entrez?Db=pubmed&Cmd=Search&Term="Izumikawa K"%5BAuthor%5D&itool=EntrezSystem2.PEntrez.Pubmed.Pubmed_ResultsPanel.Pubmed_DiscoveryPanel.Pubmed_RVAbstractPlus), [Kakeya H](http://www.ncbi.nlm.nih.gov/sites/entrez?Db=pubmed&Cmd=Search&Term="Kakeya H"%5BAuthor%5D&itool=EntrezSystem2.PEntrez.Pubmed.Pubmed_ResultsPanel.Pubmed_DiscoveryPanel.Pubmed_RVAbstractPlus), [Yamamoto Y](http://www.ncbi.nlm.nih.gov/sites/entrez?Db=pubmed&Cmd=Search&Term="Yamamoto Y"%5BAuthor%5D&itool=EntrezSystem2.PEntrez.Pubmed.Pubmed_ResultsPanel.Pubmed_DiscoveryPanel.Pubmed_RVAbstractPlus), [Tashiro T](http://www.ncbi.nlm.nih.gov/sites/entrez?Db=pubmed&Cmd=Search&Term="Tashiro T"%5BAuthor%5D&itool=EntrezSystem2.PEntrez.Pubmed.Pubmed_ResultsPanel.Pubmed_DiscoveryPanel.Pubmed_RVAbstractPlus), [Kohno S](http://www.ncbi.nlm.nih.gov/sites/entrez?Db=pubmed&Cmd=Search&Term="Kohno S"%5BAuthor%5D&itool=EntrezSystem2.PEntrez.Pubmed.Pubmed_ResultsPanel.Pubmed_DiscoveryPanel.Pubmed_RVAbstractPlus).Elevated levels of high mobility group box chromosomal protein-1 (HMGB-1) in sera from patients with severe bacterial pneumonia coinfected with influenza virus. [*Scand J Infect Dis.*](javascript:AL_get(this, 'jour', 'Scand J Infect Dis.');) 2008; 28: 338-342..
8. [Kosai K](http://www.ncbi.nlm.nih.gov/sites/entrez?Db=pubmed&Cmd=Search&Term="Kosai K"%5BAuthor%5D&itool=EntrezSystem2.PEntrez.Pubmed.Pubmed_ResultsPanel.Pubmed_DiscoveryPanel.Pubmed_RVAbstractPlus), [Seki M](http://www.ncbi.nlm.nih.gov/sites/entrez?Db=pubmed&Cmd=Search&Term="Seki M"%5BAuthor%5D&itool=EntrezSystem2.PEntrez.Pubmed.Pubmed_ResultsPanel.Pubmed_DiscoveryPanel.Pubmed_RVAbstractPlus), [Yanagihara K](http://www.ncbi.nlm.nih.gov/sites/entrez?Db=pubmed&Cmd=Search&Term="Yanagihara K"%5BAuthor%5D&itool=EntrezSystem2.PEntrez.Pubmed.Pubmed_ResultsPanel.Pubmed_DiscoveryPanel.Pubmed_RVAbstractPlus), [Nakamura S](http://www.ncbi.nlm.nih.gov/sites/entrez?Db=pubmed&Cmd=Search&Term="Nakamura S"%5BAuthor%5D&itool=EntrezSystem2.PEntrez.Pubmed.Pubmed_ResultsPanel.Pubmed_DiscoveryPanel.Pubmed_RVAbstractPlus), [Kurihara S](http://www.ncbi.nlm.nih.gov/sites/entrez?Db=pubmed&Cmd=Search&Term="Kurihara S"%5BAuthor%5D&itool=EntrezSystem2.PEntrez.Pubmed.Pubmed_ResultsPanel.Pubmed_DiscoveryPanel.Pubmed_RVAbstractPlus), [Imamura Y](http://www.ncbi.nlm.nih.gov/sites/entrez?Db=pubmed&Cmd=Search&Term="Imamura Y"%5BAuthor%5D&itool=EntrezSystem2.PEntrez.Pubmed.Pubmed_ResultsPanel.Pubmed_DiscoveryPanel.Pubmed_RVAbstractPlus), [Izumikawa K](http://www.ncbi.nlm.nih.gov/sites/entrez?Db=pubmed&Cmd=Search&Term="Izumikawa K"%5BAuthor%5D&itool=EntrezSystem2.PEntrez.Pubmed.Pubmed_ResultsPanel.Pubmed_DiscoveryPanel.Pubmed_RVAbstractPlus), [Kakeya H](http://www.ncbi.nlm.nih.gov/sites/entrez?Db=pubmed&Cmd=Search&Term="Kakeya H"%5BAuthor%5D&itool=EntrezSystem2.PEntrez.Pubmed.Pubmed_ResultsPanel.Pubmed_DiscoveryPanel.Pubmed_RVAbstractPlus), [Yamamoto Y](http://www.ncbi.nlm.nih.gov/sites/entrez?Db=pubmed&Cmd=Search&Term="Yamamoto Y"%5BAuthor%5D&itool=EntrezSystem2.PEntrez.Pubmed.Pubmed_ResultsPanel.Pubmed_DiscoveryPanel.Pubmed_RVAbstractPlus), [Tashiro T](http://www.ncbi.nlm.nih.gov/sites/entrez?Db=pubmed&Cmd=Search&Term="Tashiro T"%5BAuthor%5D&itool=EntrezSystem2.PEntrez.Pubmed.Pubmed_ResultsPanel.Pubmed_DiscoveryPanel.Pubmed_RVAbstractPlus), [Kohno S](http://www.ncbi.nlm.nih.gov/sites/entrez?Db=pubmed&Cmd=Search&Term="Kohno S"%5BAuthor%5D&itool=EntrezSystem2.PEntrez.Pubmed.Pubmed_ResultsPanel.Pubmed_DiscoveryPanel.Pubmed_RVAbstractPlus). Gabexate mesilate suppresses influenza pneumonia in mice through inhibition of cytokines. *J Int Med Res* 2008, 36: 322-328.
9. Seki M, Kosai K, Yanagihara K, Higashiyama Y, Kurihara S, Izumikawa K, Miyazaki Y, Hirakata Y, Tashiro T and Kohno S. Disease severity in patients with simultaneous influenza and bacterial pneumonia *Intern Med*, 2007, 46: 953-958.
10. SekiM, Hashiguchi K, Kosai K, Higashiyama Y, Yanagihara, K, Kurihara S, Izumikawa K, Miyazaki Y, Hirakata Y, Tashiro T and Kohno S. A patient with fulminant primary influenza pneumonia which developed into secondary bacterial pneumonia. *Acta Medica Nagasakiensia*, 2006; 51: 121-124.
11. Seki M, Yanagihara K, Higashiyama Y, Fukuda Y, Kaneko Y, Ohno H, Miyazaki Y, Hirakata Y, TomonoK, Kadota J, Tashiro T, Kohno S. Immunokinetics in severe pneumonia due to influenza virus and bacteria coinfection in mice. *Eur Respir J*., 2004; 24, 143-149.
12. [Seki M, Higashiyama Y, Tomono K, Yanagihara K, Ohno H, Kaneko Y, Izumikawa K, Miyazaki Y, Hirakata Y, Mizuta Y, Tashiro T, Kohno S.](http://www.ncbi.nlm.nih.gov/entrez/query.fcgi?db=pubmed&cmd=Retrieve&dopt=AbstractPlus&list_uids=15196241&query_hl=1&itool=pubmed_docsum) Acute infection with in_uenza virus enhances susceptibility to fatal pneumonia following Streptococcuspneumoniae infection in mice with chronic pulmonary colonization with Pseudomonas aeruginosa. *Clin Exp Immunol*., 2004; 137, 35-40.
13. Imamura Y, Yanagihara K, Mizuta Y, Seki M, Ohno H, Higashiyama Y, Miyazaki Y, Tsukamoto K,Hirakata Y, Tomono K, Kadota J, Kohno S. Azithromycin inhibits MUC5AC production induced by the Pseudomonas aeruginosa autoinducer N-(3-Oxododecanoyl) homoserine lactone in NCI-H292 Cells. *Antimicrob Agents Chemother*.2004; 48,3457-3461.
14. Kido H, Okumura Y, Yamada H, Mizuno D, Higashi Y, Yano M. [Secretory leukoprotease inhibitor and pulmonary surfactant serve as principal defenses against influenza A virus infection in the airway and chemical agents up-regulating their levels may have therapeutic potential.](http://www.ncbi.nlm.nih.gov/pubmed/15576322?ordinalpos=2&itool=EntrezSystem2.PEntrez.Pubmed.Pubmed_ResultsPanel.Pubmed_DefaultReportPanel.Pubmed_RVDocSum) *Biol Chem*. 2004; 385: 1029-34.
15. Sato K, Suga M, Akaike T, Fujii S, Muranaka H, Doi T, Maeda H, Ando M. [Therapeutic effect of erythromycin on influenza virus-induced lung injury in mice.](http://www.ncbi.nlm.nih.gov/pubmed/9517602?ordinalpos=12&itool=EntrezSystem2.PEntrez.Pubmed.Pubmed_ResultsPanel.Pubmed_DefaultReportPanel.Pubmed_RVDocSum) *Am J Respir Crit Care Med.* 1998;157: 853-7.
16. [McCullers JA](http://www.ncbi.nlm.nih.gov/pubmed?term="McCullers JA"%5BAuthor%5D&itool=EntrezSystem2.PEntrez.Pubmed.Pubmed_ResultsPanel.Pubmed_RVAbstract). Effect of antiviral treatment on the outcome of secondary bacterial pneumonia after influenza. [*J Infect Dis.*](javascript:AL_get(this, 'jour', 'J Infect Dis.');) 2004;190: 519-26.
17. Laurent Kaiser, R. Scott Fritz, Stephen E. Straus, Larisa Gubareva, and Frederick G. Hayden. Symptom Pathogenesis During Acute Infuenza: Interleukin-6 and Other Cytokine Responses. *Journal of Medical Virology* , 2001: 64:262-268
18. ペラミビル水和物　公開審査資料概要；医薬品医療機器総合機構ホームページ、新薬の承認審査に関する情報より
